# Prospective Evaluation of Feline Sourced Platelet-Rich Plasma Using Centrifuge-Based Systems

**DOI:** 10.3389/fvets.2020.00322

**Published:** 2020-06-12

**Authors:** Jonathan T. Ferrari, Pamela Schwartz

**Affiliations:** Department of Surgery, The Animal Medical Center, New York, NY, United States

**Keywords:** platelet-rich plasma, platelet count, feline regenerative medicine, platelet aggregation, platelet clumping

## Abstract

**Objective:** To evaluate the hematologic components of platelet-rich plasma (PRP) generated using feline blood with two commercially available centrifuge-based systems^1^^,^^2^.

**Materials and methods:** Twenty healthy adult cats were enrolled in this prospective study from November 2018 to January 2019. Feline blood samples were obtained for analysis of whole blood (WB) cellular components and preparation of PRP product. PRP was prepared using two commercial systems and complete blood count (CBC) testing was performed on both WB and PRP samples. The cellular composition of the PRP product was compared to the WB sample for each patient.

**Results:** Both systems showed significant decrease of median RBC concentration in PRP products compared to WB samples (*P* = 0.002 for both systems). System 1 significantly decreased median WBC concentration (*P* = 0.002). System 2 decreased WBC concentration, though statistical significance was not reached (*P* = 0.63). Median platelet concentration was decreased by 3% using System 1, and increased by 187% using System 2. Platelet aggregation presented a challenge with 8/20 (40%) of samples demonstrating platelet aggregation.

**Clinical relevance:** Commercial systems available for generation of PRP may be useful for creating a feline sourced product and in this study showed promise in decreasing RBC and WBC concentration. Neither system tested achieved 2–5 times platelet concentration from baseline. Platelet aggregation presented a significant obstacle to reliable generation of PRP products using feline blood. This treatment modality may be particularly beneficial for feline patients with osteoarthritis and soft tissue injuries, though first characterizing the PRP product made using feline blood is critical to validate its use in further clinical studies.

## Introduction

Platelet-rich plasma (PRP) is defined as a blood concentrate with a higher concentration of platelets than whole blood (WB) (1). In addition to their hemostatic action, platelets function in wound healing by releasing alpha granules containing growth factors which initiate cellular migration, angiogenesis, and matrix deposition (2–7). Studies involving PRP use for human, equine, and canine patients with orthopedic, and soft tissue injuries have demonstrated improved function and perceived pain control in the settings of osteoarthritis and tendinopathies (8–18). Canine sourced PRP has also been demonstrated to improve the gross and histologic rate of wound healing, confer antibiotic properties for cutaneous wound healing, and improve survival of skin flaps (19–24). The use of PRP for management of musculoskeletal or soft tissue injuries has not been investigated in feline patients.

For a blood product to be categorized as PRP, the platelet concentration must be > 1,000 K/uL or within a range of 2–5 times that of baseline WB concentration (1, 25). PRP also contains red blood cells (RBC) and white blood cells (WBC), and the ideal concentration of these components for therapeutic effect is disputed. PRP function may be enhanced by the presence of leukocytes, though some authors argue that neutrophils can induce inflammation causing tissue damage (26, 27). An inhibitory effect on osteoblast and fibroblast proliferation has also been described with higher platelet concentration in PRP samples (28). Thus, the variability in PRP product composition presents a challenge to reliable generation and clinical efficacy. Validation of the product composition prior to clinical use is important.

Multiple commercial systems are available for generation of PRP and prior studies have previously evaluated the cellular composition of PRP generated using canine blood (29–31). These studies show inconsistent concentrations of platelets and WBC in PRP products across commercially available systems (29–31). A recent study evaluating five different systems using canine blood found differing concentration of platelets, neutrophils, and RBC between systems (31). Despite the reported discrepancies in composition across systems, clinical studies using PRP in canine patients have shown benefit in osteoarthritis, wound healing, and tendinopathies (8–24).

Given the clinical benefit of PRP in human, equine, and canine patients, it is reasonable to suspect that this therapy may be similarly efficacious for feline patients. While prior reports have demonstrated success producing feline platelet concentrate (32–34), evaluation of commercially available systems has not been reported. To the authors' knowledge, the only reported use of PRP in cats is that of a canine sourced PRP applied to a cutaneous wound of a feline patient (35). Prior work has shown promise using meloxicam, tramadol, and recently a feline-specific anti-nerve growth factor monoclonal antibody in cats with osteoarthritis (36–40). However, these medical options may be limited in some feline patients. Non-steroidal anti-inflammatory drugs are often contraindicated in geriatric cats with osteoarthritis due to concurrent chronic kidney disease. Some cats may develop dose-dependent side effects of tramadol use (36). Therefore, use of a feline sourced PRP may be particularly beneficial for patients with osteoarthritis and soft tissue injuries. First characterizing the PRP product made using feline blood is critical to validate its use.

The hypothesis of this study was that PRP products generated using the commercial systems would demonstrate concentration of platelets at least two times that of WB. A secondary goal of this study was to determine the concentrations of RBC and WBC components of the PRP products in comparison to baseline WB values.

## Materials and Methods

A prospective study was performed from November 2018 through January 2019 at the Animal Medical Center Elmer and Mamdouha Bobst Hospital. In this study all feline participants were employee-owned adult cats without a history of neoplasia, anemia (hematocrit <30%), or concurrent use of corticosteroid or other immunosuppressive therapeutics. For inclusion in the study, cats were required to be at least 1 year of age and weigh > 3.0 kg to ensure safety when collecting the required blood volume. All animals were deemed healthy by veterinarian physical examination, CBC, and serum biochemistry panel. Informed owner consent was obtained prior to inclusion in the study, and the study protocol was approved by the Institutional Animal Care and Use Committee. All patients were supervised by a veterinarian during participation in this study.

Two commercial systems were analyzed as a part of this study^1, 2^ and patients were randomly assigned to a system. All cats were sedated to achieve a plane of sedation suitable for jugular venipuncture using dexmedetomidine^3^. Jugular venipuncture was performed using a sterile 21-gauge-butterfly needle for blood sample acquisition. Following sample collection, sedation was reversed using an appropriate intramuscular dose of atipamezole^4^. Sample collection and preparation was performed according to manufacturer recommendations, with the only modification being a limitation of the blood volume obtained to account for the lower body weight of feline patients compared to canine patients. To ensure that the blood obtained from any feline patient was <10% of the individual's blood volume, venipuncture was restricted to 16 mL for System 1 and 15 mL for System 2. Blood samples (2.5 mL) were first collected for complete blood count (CBC) and placed into standard ethylenediaminetetraacetic acid (EDTA) collection tubes^5^, and samples were then collected for PRP generation during the same venipuncture event using manufacturer syringes. Anticoagulant citrate dextrose solution A^6^ (ACD-A) was added to each syringe prior to sample collection as instructed by each manufacturer.

**Figure 1:**
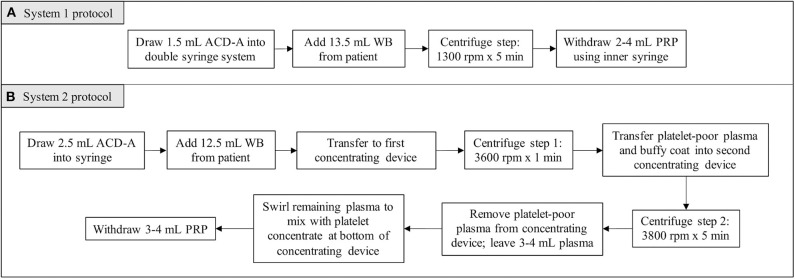
Protocol for generation of feline PRP using System 1 **(A)** and System 2 **(B)**.

For System 1, 1.5 mL of ACD-A and 13.5 mL of patient blood were added to the manufacturer syringe. The volumes of ACD-A and blood were within the range recommended by the manufacturer for canine patients. System 1 utilized a double-syringe system and a single centrifugation step (1,300 rpm × 5 min), after which the PRP was withdrawn into the inner syringe (Figure 1A).

For System 2, 2.5 mL of ACD-A and 12.5 mL of patient blood were added to a syringe. For canine patients, the manufacturer recommended larger volumes of ACD-A and blood. In restricting the volume of blood obtained from each patient to account for the lower body weight of feline patients, the volume of ACD-A was restricted as well while maintaining the ratio recommended by the manufacturer. The blood mixed with ACD-A was transferred into the first concentrating device for the first centrifugation step (3,600 rpm × 1 min). Following the first centrifugation step, the platelet and plasma buffy coat was collected into a new syringe and transferred into the second concentrating device. The second centrifugation step was then performed (3,800 rpm × 5 min), after which the platelet-poor plasma product was withdrawn from the concentrating device and discarded. The platelet concentrate was resuspended in the remaining plasma by gently swirling the concentrating device, and the resultant PRP was collected (Figure 1B).

Baseline CBC was performed including WBC differential, RBC concentration, and platelet concentration using an in-house hematology analyzer^7^. The same analyzer was used immediately following sample preparation to assess the PRP product. The WB and PRP product RBC, WBC, neutrophil, lymphocyte, monocyte, and platelet concentrations were determined for each sample. Data were non-normal in all except RBC (Shapiro Wilk test performed). Therefore, non-normal data were analyzed by means of the non-parametric Wilcoxon rank sum test for the unpaired comparisons of System 1-System 2 and the non-parametric Wilcoxon signed rank test for the paired comparisons of WB-PRP. Results were reported as median with lower (25th) and upper (75th) quartiles. These values were analyzed using statistical software^8^ and *P*-values were reported with significance set at *P* < 0.05.

## Results

Twenty cats were enrolled in this study, with breeds represented including Domestic Shorthair (*n* = 18) and Domestic Longhair (*n* = 2) breeds. Of these, nine were neutered males and 11 were spayed females. Mean age of patients was 5.7 years (range 1–15 years) and mean weight was 5.6 kg (range 3.43**–**8.9 kg).

Ten blood samples were used for each system studied; patients were randomly assigned to a group and no patient sample was used for both groups. Group 1 was tested using System 1 and consisted of 10 Domestic Shorthair cats, and five each were female spayed and male neutered cats. The median weight of patients in group 1 was 5.35 kg (range 3.4–8.9 kg) and the median age was 5 years (range 1–15). Group 2 was tested using System 2 and consisted of eight Domestic Shorthair and two Domestic Longhair cats, and six were female spayed and four were male neutered cats. The median weight of patients in group 2 was 5.25 kg (range 4.4–8.3 kg) and the median age was 7 years (range 1–10).

Clumping of platelets occurred in the WB samples of 8/20 (40%) cats; three of these samples were in the System 1 group and five were in the System 2 group. For the purposes of statistical analysis, these patients were included in analyses for concentration of RBC, neutrophils, lymphocytes, and monocytes, but excluded from statistical analysis for platelet concentration.

### System 1

The volume of PRP generated using System 1 ranged from 2 to 4 mL per patient. Median RBC and WBC concentrations were both significantly decreased compared to the WB samples. Median RBC concentration was decreased by 99.9% (*P* = 0.002). Median concentration of each WBC component (neutrophils, lymphocytes, monocytes) was decreased by 100% (*P* = 0.002). Platelet clumping was documented of the WB samples for 3/10 (30%) samples. The samples with platelet clumping were excluded from analysis for platelet concentration. The median platelet concentration was decreased by 3% compared to the WB samples, and this was not statistically significant (*P* = 0.98) (Table 1).

**Table 1:** Median (quartile 1**—**quartile 3) values of cellular components of WB and PRP products generated using two commercial systems.

	**WB**	**PRP**	***P-*value**
**System 1**			
RBC (K/μL)	8.95 (9.59–8.15)	0.12 (0.07–0.18)	0.002
WBC (K/μL)	9.65 (8.00–10.80)	0.00	0.002
Neutrophils (K/μL)	6.08 (3.42–7.31)	0.00	0.002
Lymphocytes (K/μL)	2.84 (2.00–4.28)	0.00	0.002
Monocytes (K/μL)	0.31 (0.29–0.35)	0.00	0.004
Platelets (K/μL)	243 (193–349)	238 (225–297)	0.98
**System 2**			
RBC (K/μL)	9.63 (8.70–9.97)	0.57 (0.28–1.78)	0.002
WBC (K/μL)	6.95 (6.50–8.60)	1.35 (0.40–15.10)	0.92
Neutrophils (K/μL)	4.47 (3.71–5.45)	0.60 (0.16–6.54)	0.63
Lymphocytes (K/μL)	2.09 (1.80–2.23)	0.60 (0.19–6.93)	0.63
Monocytes (K/μL)	0.24 (0.20–0.28)	0.08 (0.02–1.06)	0.63
Platelets (K/μL)	269 (239–291)	505 (166–1094)	0.44

*N = 10 for all, except for platelet analysis, for which n = 7 for System 1 and n = 5 for System 2 due to sample exclusion when affected by platelet clumping detected in the WB samples. Comparisons were paired non-parametric by means of the Wilcoxon signed rank test (WSRT). P-values were reported with significance set at P < 0.05*.

### System 2

The volume of PRP made using System 2 ranged from 3 to 4 mL per patient. Median RBC concentration was significantly decreased compared to the WB samples by 94% (*P* = 0.002). Median WBC concentration was decreased by 80% and this difference was not statistically significant (*P* = 0.92). When comparing the PRP product to the WB samples, neutrophil concentration was decreased by 86.6% (*P* = 0.63), lymphocyte concentration was decreased by 71% (*P* = 0.63), and monocyte concentration was decreased by 66.6% (*P* = 0.63). Platelet clumping was documented of the WB samples for 5/10 (50%) of the samples. The samples with platelet clumping were excluded from analysis for platelet concentration. The median platelet concentration of the included PRP products was increased by 187% compared to the WB samples, and this was not statistically significant (*P* = 0.44) (Table 1).

## Discussion

In this study, both systems reduced the median RBC concentration of the PRP products compared to WB samples. However, neither system concentrated platelets by 2–5 times baseline, or had platelet concentration > 1,000 K/uL, which is ideal for a PRP product (1, 25). Median platelet concentration was decreased by 3% using System 1, while System 2 increased the median platelet concentration by 187%. Therefore, the use of the term “PRP” to describe these products may be inappropriate. For the purpose of consistency and clarity, this discussion will continue to use the term “PRP,” though the validity of characterizing these products as such is in question.

System 1 completely excluded monocytes, neutrophils, and lymphocytes. However, this system did not result in an increase in median platelet concentration. System 1 required only one step of centrifugation, eliminating a second step of centrifugation during preparation that is common in other PRP systems. It is possible that for feline blood, the second centrifugation step may important for platelet concentration, or that centrifuge settings should be altered as those used in this study were those recommended for canine blood. This may represent a species difference between the feline samples tested in this study and canine blood samples for which this system was manufactured. As this study did not assess the clinical efficacy of these products, it is unknown if the PRP products generated using System 1, which had similar platelet concentrations to the WB samples with significantly reduced RBC and WBC concentration, would have applications *in vivo*.

System 2 increased median platelet concentration in the PRP samples (187% increase), though this concentrate falls short of the ideal concentration 2–5 times the baseline. Several samples (5/10) were excluded due to platelet clumping detected in the WB, perhaps resulting in type II error. It is possible that the median platelet concentration may be higher if fewer samples demonstrated platelet clumping and could have been included in analysis. System 2 demonstrated 80% reduction of leukocyte concentration, which characterizes this product as leukocyte-poor PRP. This data demonstrating nearly 2-fold platelet concentration is promising for the potential clinical use of this product.

A major finding of this study was platelet clumping documented in 8/20 (40%) WB samples. The samples that clumped were excluded from statistical analysis for platelet concentration, which resulted in smaller sample sizes. Platelet aggregation is a common problem resulting in pseudothrombocytopenia in feline hematologic laboratory samples (32). As platelet aggregation occurred in both groups, it is suspected that platelet aggregation was due to species-specific factors or collection method. In the present study, WB samples for CBC processing were collected first and placed within standard EDTA-collection tubes, followed by blood collection into syringes for PRP preparation with ACD-A pre-loaded in the syringe as anticoagulant. The anticoagulant used here (EDTA for WB sample for baseline CBC; ACD-A for PRP product preparation) may not be appropriate for feline blood in this application. It has been previously suggested that EDTA rather than citrate should be used for anticoagulation of canine samples, and other anticoagulants including iloprost have been recommended for feline blood samples (34, 41, 42). Another consideration may be applying ACD-A to the sterile line used for venipuncture, thereby admixing the WB samples with anticoagulant within the line prior to entry into the tube or syringe. Further investigation is warranted to determine the best method to prevent feline platelet aggregation.

This study was limited by small sample size, which may have resulted in type II error. Manual platelet counting was not included in this study and may be a method to avoid eliminating samples due to platelet aggregation affecting instrument counting. In addition, the method of feline platelet counting was not validated and it has been suggested that hematologic analyzers be validated specifically for PRP samples to ensure accurate platelet counting (43).

The study reported here was designed to evaluate the ability of commercial systems to concentrate platelets using feline blood, as well as to evaluate the other cellular components of the PRP products. The data show that the cellular composition of PRP products from the systems differed in important ways. System 1 did not result in an increased concentration of platelets. System 2 showed an increase in the median platelet concentration, though not to a degree to meet the standard definition of PRP. These results underscore the need for standardization and validation of these systems if clinical efficacy is to be expected. Conclusions regarding the clinical use of the PRP products cannot be drawn from this study. Further study is necessary for evaluation of clinical efficacy of PRP use in feline patients.

## Data Availability Statement

The datasets generated for this study are available on request to the corresponding author.

## Ethics Statement

The animal study was reviewed and approved by Institutional Animal Care and Use Committee of the Animal Medical Center. Written informed consent was obtained from the owners for the participation of their animals in this study.

## Author Contributions

Both authors assisted with study development, procedures, and manuscript preparation.

## Conflict of Interest

Centrifuge and regenerative medicine products were provided by Arthrex^1^ for the purposes of this study. No other third-party funding or support was received in connection with study design, testing, data analysis, or manuscript preparation. The authors declare that the research was conducted in the absence of any commercial or financial relationships that could be construed as a potential conflict of interest.

## Footnotes

^3^Dexmedetomidine hydrochloride, Zoetis, Kalamazoo, MI.

^4^Atipamezole hydrochloride, Zoetis, Kalamazoo, MI.

^5^Becton, Dickinson and Company, Franklin Lakes, NJ.

^6^Anticoagulant citrate dextrose solution, solution A, Citra Labs, Braintree, ME.

^7^Idexx LaserCyte Hematology Analyzer, Idexx Laboratories, Westbrook, ME.

^8^SAS v. 9.3, SAS Institute Inc, Cary, NC.

## Abbreviations

ACD-A: Anticoagulant citrate dextrose solution A
CBC: Complete blood count
EDTA: Ethylenediaminetetraacetic acid
PRP: Platelet-rich plasma
RBC: Red blood cell
WBC: White blood cell
WB: Whole blood.
